# Continuous reorganization of cortical information flow in multiple sclerosis: A longitudinal fMRI effective connectivity study

**DOI:** 10.1038/s41598-020-57895-x

**Published:** 2020-01-21

**Authors:** Vinzenz Fleischer, Muthuraman Muthuraman, Abdul Rauf Anwar, Gabriel Gonzalez-Escamilla, Angela Radetz, René-Maxime Gracien, Stefan Bittner, Felix Luessi, Sven G. Meuth, Frauke Zipp, Sergiu Groppa

**Affiliations:** 1 Department of Neurology, Focus Program Translational Neuroscience (FTN), University Medical Center of the Johannes Gutenberg University, Mainz, Germany; 2 Bio-medical Engineering Department, University of Engineering & Technology, Lahore, Pakistan; 3 Department of Neurology and Brain Imaging Center, Goethe University, (Main), Frankfurt, Germany; 4 Department of Neurology with Institute of Translational Neurology, University of Münster, Münster, Germany

**Keywords:** Neuroscience, Multiple sclerosis

## Abstract

Effective connectivity (EC) is able to explore causal effects between brain areas and can depict mechanisms that underlie repair and adaptation in chronic brain diseases. Thus, the application of EC techniques in multiple sclerosis (MS) has the potential to determine directionality of neuronal interactions and may provide an imaging biomarker for disease progression. Here, serial longitudinal structural and resting-state fMRI was performed at 12-week intervals over one year in twelve MS patients. Twelve healthy subjects served as controls (HC). Two approaches for EC quantification were used: Causal Bayesian Network (CBN) and Time-resolved Partial Directed Coherence (TPDC). The EC strength was correlated with the Expanded Disability Status Scale (EDSS) and Fatigue Scale for Motor and Cognitive functions (FSMC). Our findings demonstrated a longitudinal increase in EC between specific brain regions, detected in both the CBN and TPDC analysis in MS patients. In particular, EC from the deep grey matter, frontal, prefrontal and temporal regions showed a continuous increase over the study period. No longitudinal changes in EC were attested in HC during the study. Furthermore, we observed an association between clinical performance and EC strength. In particular, the EC increase in fronto-cerebellar connections showed an inverse correlation with the EDSS and FSMC. Our data depict continuous functional reorganization between specific brain regions indicated by increasing EC over time in MS, which is not detectable in HC. In particular, fronto-cerebellar connections, which were closely related to clinical performance, may provide a marker of brain plasticity and functional reserve in MS.

## Introduction

Multiple sclerosis (MS) is a chronic immune-mediated disease of the central nervous system characterized by demyelination, inflammation and neurodegeneration^1^. Whereas conventional MRI is very sensitive for the detection of demyelinating lesions as well as for the quantification of brain atrophy, functional MRI (fMRI) is able to examine the resultant changes in connectivity by measuring the temporal dependency of neuronal activation patterns of anatomically separated brain regions^2^. Investigations of such connectivity patterns in MS patients have demonstrated that functional reorganization processes are important for counteracting continuous damage resulting from demyelination and neuronal loss^3–5^.

Effective connectivity (EC) estimations as derived from fMRI allow quantification of information flows in neural networks. While functional connectivity describes statistical dependencies between spatially segregated neuronal activities, and structural connectivity the morphological correlate, EC can be analysed to estimate directed coupling of information flow^6^. Hence, EC is able to explore causal effects between cortical areas, which are highly relevant for biological network behaviour and can be traced longitudinally to depict brain reorganization processes in brain diseases^7,8^.

Early EC studies in MS patients demonstrated higher EC levels during Paced Auditory Serial Addition Test (PASAT) performance in the working memory network^9^, during a sensorimotor and information processing speed task within frontal networks^10,11^ as well as during Stroop task performance in the sensorimotor cortex^10^ - each in a cross-sectional approach. These differences in EC measures between MS patients and healthy controls (HC) were primarily interpreted as an adaptive response to maintain cognitive and/or sensorimotor function for review see^12^. Recently, altered connectivity patterns were found during high cognitive control demands in the executive control network among different MS phenotypes^13^, in particular a loss of top-down connections was seen in patients with progressive MS^14^.

These task-related EC changes point towards dynamic connectivity patterns that change during the course of the disease. But in contrast to task-dependent approaches, the dynamics of EC in the absence of behavioural measures are largely unknown and existing research still lacks the understanding of how directed connectivity changes over time in MS patients. Hence, longitudinal investigations of brain reorganization in MS patients are necessary to provide maps of brain plasticity processes over time, very likely relevant for disability progression.

In our study, we performed two parallel EC analyses based on recently established methods, neither of which require any a priori information on the existence of a connection between two regions as is necessary for dynamic causal modelling^10,15^ or structural equation modelling (SEM)^9,16^. Specifically, we analysed EC based on Causal Bayesian Networks (CBN) proposed by Smith *et al*.^17^ and, since EC can also be simultaneously estimated in two domains (namely frequency and time), we additionally assessed EC based on the so-called Time-resolved Partial Directed Coherence (TPDC) method^18–23^ developed in the context of electrophysiological measurements of frequency-filtered blood oxygen-level dependent (BOLD) fMRI time courses.

Ultimately, we aimed to provide a longitudinal outline of EC changes derived from resting-state fMRI with these two methodological approaches. Therefore, relapsing-remitting (RR)MS patients were followed-up over five successive time points at twelve-week intervals. In addition, we validated these findings by comparing our results with longitudinal EC data from HC over the same period of time. Finally, we investigated the link between the strength of EC and clinical disability, patient fatigue and disease duration.

## Patients and Methods

### Subjects

Subjects were recruited at the Department of Neurology, University Medical Center of the Johannes Gutenberg University Mainz and at the Department of Neurology, Goethe-University Frankfurt, and measured with a standardized MRI protocol in Mainz^24^. Healthy subjects served as controls (HC). Written informed consent was obtained from all subjects before participation in this study, which was approved by the Ethics Committee of the State Medical Association of Rhineland-Palatine. The study has been conducted according to the principles expressed in the Declaration of Helsinki. Each MS patient was clinically assessed by an experienced neurologist and the Expanded Disability Status Scale (EDSS) score was determined at study entrance and after one year. Fatigue was classified on the basis of the Fatigue Scale for Motor and Cognitive functions (FSMC)^25^ and was also gathered at study entrance and after one year. All patients had been relapse-free for at least 60 days prior to entering the study. To ensure a clinically homogenous set of patients, we decided to exclude those patients with clinical relapses during the study period.

### MRI data acquisition – longitudinal functional MRI

Measurements were performed on a 3 Tesla scanner (Magnetom Tim Trio, Siemens Healthcare, Erlangen, Germany) with a 32-channel receive-only head coil.

Functional MRI (fMRI) data were obtained using a gradient echo (GE)-EPI sequence (TR = 3000 ms, TE = 30 ms, flip angle = 90°, field of view (FOV) = 192 × 192 mm², matrix size = 64 × 64, spatial in-plane resolution: 3 mm, 49 slices with a slice thickness of 2 mm and an inter-slice gap of 1 mm, readout bandwidth (BW) = 2232 Hz/pixel). A series of 200 volumes were acquired, resulting in scan duration of 10 min^26^. The fMRI resting-state data were acquired every 12 weeks over 12 months for a total of five time points for each patient.

Pre-processing of fMRI scans and time series extraction were performed using the statistical parametric mapping toolbox (SPM8; http://www.fil.ion.ucl.ac.uk/spm). For each subject, realignment was first performed to remove movement related artifacts (e.g. head motion) in fMRI time series. In order to realign, the first image from the recording was specified as the reference image and all subsequent images were realigned to it. Scans were then spatially normalized to align all the subjects’ specific MR sequences into the standard Montreal Neurological Institute (MNI) space. The scans were smoothed by convolving them with a Gaussian kernel of fixed width (full width half maximum 8 × 8 × 8 mm) to suppress noise and effects due to differences in anatomy^20^.

After preprocessing, the time series extraction of all 116 AAL (Automated Anatomical Labeling) regions of interest (ROIs) was carried out using the CONN toolbox^27^. To gain a conceptual and robust overview of EC changes of the entire brain, we avoided using a small compartmentalized brain atlas and rather selected large-scale ROIs in light of maximal population-level reproducibility and biological validity while also accommodating individual anatomic variability. Hence we merged the 116 ROIs of the AAL atlas into seven superordinate regions, namely prefrontal (including 4 AAL-based brain structures), frontal (including 22 AAL-based brain structures), parietal (including 20 AAL-based brain structures), occipital (including 14 AAL-based brain structures), temporal (including 22 AAL-based brain structures), deep grey matter nuclei (DGMN; including 8 AAL-based brain structures) and cerebellum (including 26 AAL-based brain structures)^28^. Supplementary Table S1 offers a detailed overview over the seven merged brain regions and their comprising AAL-based subregions. The time series were pooled according to the low frequency fluctuations amplitude in each of these seven regions, ranging from 0.009–0.08 Hz, e.g.^20,29^.

**Figure 1:**
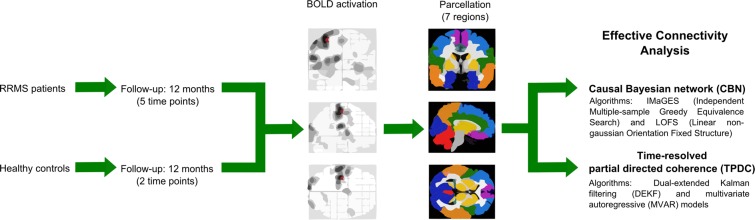
Workflow diagram. Preprocessing step was performed using the statistical parametric mapping toolbox (SPM8). After preprocessing, the time series extraction of all 116 ROIs of the AAL atlas was carried out using the CONN toolbox. The images shown under “parcellation” demonstrate the division of the initial 116 ROIs into seven merged regions, namely prefrontal, frontal, parietal, occipital, temporal, deep grey matter nuclei and cerebellum. Subsequently, we estimated effective connectivity (EC) strength using Causal Bayesian Networks (CBN) and Time-resolved Partial Directed Coherence (TPDC).

The complete workflow is shown in Fig. 1.

### MRI data acquisition – structural MRI volumetric analysis

We also calculated brain volumes. For this, imaging was performed using a sagittal 3D T1-weighted magnetization prepared rapid gradient echo imaging (MP-RAGE) sequence (TE/TI/TR = 2.52/900/1900 ms, flip angle = 9°, field of view (FOV) = 256 × 256 × 192 mm^3^, matrix size = 256 × 256 × 192, voxel size = 1 × 1 × 1 mm^3^ BW = 170 Hz/pixel) and sagittal 3D T2-weighted fluid attenuated inversion recovery (FLAIR) sequence (TE/TI/TR = 388/1800/5000 ms, echo-train length = 848, FOV = 256 × 256 mm, matrix size = 256 × 256, slab thickness = 192 mm, voxel size = 1 × 1 × 1 mm). Tissue abnormalities were excluded by a specialized neuroradiologist. For the VBM analysis, SPM8 with the lesion segmentation toolbox (LST) (http://www.applied-statistics.de/lst.html) and VBM8 toolbox (http://dbm.neuro.uni-jena.de/vbm/) were used. Lesion maps were drawn manually on T2-weighted 3D FLAIR images using the MRIcron software (http://www.mccauslandcenter.sc.edu/mricro/mricron/). First, using LST, 3D FLAIR images were co-registered to 3D T1 images and bias corrected. After partial volume estimation (PVE), lesion segmentation was performed with 20 different initial threshold values for the lesion growth algorithm^30^. By comparing automatically and manually estimated lesion maps, the optimal threshold (ĸ value, dependent on image contrast) was determined for each patient and an averaged value for all patients calculated. Afterwards, for automatic lesion volume estimation and filling of 3D T1 images, a uniform ĸ value of 0.1 was applied in all patients. Subsequently, the filled 3D T1 images as well as the native 3D T1 images were segmented into grey matter (GM), white matter (WM), and cerebrospinal fluid (CSF) and normalized to MNI space.

### Effective connectivity analysis

#### Causal bayesian network (CBN) analysis

The first EC analysis was performed based on the seven predefined regions using the CBN method^31^. Two algorithms, namely IMaGES and the LOFS with Tetrad software (Version 5.6), were applied^32,33^. This analysis provides the direction of temporal influence among the regions within the network as well as the strength of the connection between regions^33^. Since CBN is search-based, there is no need for an a priori model of connections, and since the search is constrained to predefined regions and penalized for overfitting, the risk of false-positive connections is reduced. First, time series from the selected 7 ROIs in the resting-state were extracted from all study subjects. Then, time series from each cohort (i.e., HC and MS separately) were used for the estimation of the connectivity values. Initially, the method starts with an empty graph and searches forward, one new connection at a time, until it finds the set of connections that optimally represents the entire group of subjects. This is done by selecting the model with the highest Bayesian Information Criterion (BIC) score across datasets^32,33^. After that, IMaGES algorithm identified a directed acyclic graph for the seven regions, then the LOFS algorithm^33^ was used to determine the dominant direction (i.e., the causality) of the connection between two regions. Finally, after the connections were detected and oriented, a SEM estimator was used to calculate the goodness of fit of each dataset to the outcome connectivity model of the LOFS algorithm. The parameter values of the SEM model (representing connection strengths for each subject in a cohort) were estimated by using a regression optimizer and used for statistical analysis^34^.

#### Time-resolved partial directed coherence (TPDC) analysis

The second EC analysis was performed using TPDC analysis. Using time-frequency causality, we cannot only focus on a particular frequency, but also analyse the dynamics of the causality at that frequency. The time-frequency causality estimation method TPDC is based on dual-extended Kalman filtering (DEKF)^35,36^, and allows time-dependent autoregressive coefficients to be estimated. One extended Kalman filtering (EKF) estimates the states and feeds this information to the other; the second EKF estimates the model parameters and shares this information with the first. By using two Kalman filters working in parallel with one another, we can estimate both states and model parameters of the system at each time instant (referred to as DEKF). After estimating the time-dependent multivariate autoregressive (MVAR) coefficients, the next step is to use those coefficients for the calculation of causality between the time series. By calculating the time-dependent MVAR coefficients at each time point, we can also calculate partial directed coherence (PDC) at each time point. The general expression for the PDC is given as follows:

1 $$|{\pi }_{i\leftarrow j}(\omega )|=\frac{|{A}_{ij}(\omega )|}{\sqrt{{\sum }_{k}{|{A}_{kj(\omega )}|}^{2}}}$$

In Eq. (1), ${\pi }_{i\leftarrow j}(\omega )$ is the magnitude of PDC from time series ${x}_{j}$ to ${x}_{i}$ at frequency $\omega$. *A*_*ij*_ are the Fourier coefficients of the causal coefficients ${a}_{ij}$. Afterwards, a time-frequency plot using all PDCs can be produced. The precise distribution of the MVAR coefficients is not known; we used a surrogate method to check for the significance of the results. In short, we divide the original time series into smaller non-overlapping windows and randomly shuffle the order of these windows to create a new time series. The MVAR model is fitted to the shuffled time series and TPDC is estimated. The shuffling process is done 1000 times and the 95^th^ percentile TPDC value is taken as the significance threshold for all of our connections. This process is performed separately for each subject^21^.

### Number of total connections

The overall region-to-region connectivity (mean EC) was estimated by taking the CBN and TPDC amplitudes between the seven merged ROIs extracted from the AAL atlas^28^, namely prefrontal, frontal, parietal, occipital, temporal, deep grey matter nuclei and cerebellum. The separation was implemented by taking all directed connections in each ROI for each of the five time points in MS patients (two time points in HC) separately. The mean CBN and TPDC values were compared between the time points with repeated-measures ANOVA (see details below).

### Statistical analysis

All statistical analyses were performed using IBM SPSS Statistics, Version 22.0 (SPSS, Chicago, Illinois, USA). Means and standard deviations (SD) as well as medians with ranges were calculated. Demographic characteristics and baseline MRI volumes of MS patients and HC were compared using Pearson’s chi-square test for categorical data and Mann-Whitney test for ordinal data and when the assumptions of a t-test were not met. To compare the EDSS and FSMC scores over time between baseline (t_1_ = 0 months) and the last follow-up (t_5_ = 12 months) we used the Wilcoxon signed-rank test. In addition, using one-way repeated measures ANOVA, the GM, WM and brain parenchymal (WM and GM) fraction as well as T2 WM lesion volume of MS patients were tested for differences between the five scanning time points.

Next, the longitudinal within-group comparison of EC values between the five time points in the group of MS patients was performed with two-way repeated measures ANOVA. Here, we aimed to assess the main effect of the two independent variables: “region” (7 regions) and “time” (5 time points). In the next step, we performed three-way repeated measures ANOVA to compare the longitudinal EC changes between MS patients and the group of HC (each between the first and last time point, as HC were only scanned twice over one year). Hence, in this repeated measures ANOVA, we aimed to assess the main effect of three independent variables: “region” (7 regions), “time” (2 time points) and “group” (2 groups). Post-hoc tests with Bonferroni’s correction for multiple comparisons were applied for all ANOVAs. The significance level was set to p < 0.05.

Finally, to investigate the link between EC and clinical impairment of the MS patients, the change of EC values (calculated as ΔEC = EC after 12 months [t_5_] – EC at baseline [t_1_]) was correlated with the EDSS, FSMC and disease duration. Significance thresholds for these correlation coefficients were adjusted for multiple comparisons by Bonferroni’s correction.

## Results

### No change in clinical impairment over time

Overall, twelve RRMS patients (7 females and 5 males; mean age 41.7 ± 11.5 years; median EDSS 1.5 [0–2.5]) completed the study relapse-free and were included in the analysis. The mean disease duration was 35.8 ± 71.3 months. In addition, twelve HC (4 females and 8 males; mean age 33.5 ± 9.6 years) served as controls and were studied longitudinally over one year at two time points. Clinical, demographic and MRI baseline data is presented in Table 1.

**Table 1:** Clinical and demographic data as well as brain volumetric measurements of relapsing-remitting multiple sclerosis (RRMS) patients and healthy controls (HC) at the baseline MR scan.

Clinical data	MS (n = 12)	HC (n = 12)	*p-value*
*Gender (female/male)*	7/5	4/8	*0.414*^(a)^
*Mean age at baseline MRI (SD) [years]*	41.7 (11.5)	33.5 (9.6)	0.078^(b)^
*Mean age at disease onset (SD) [years]*	38.3 (12.8)	—	—
*Mean disease duration (SD) [months]*	35.8 (71.3)	—	—
*Median EDSS (range)*	1.5 (0–2.5)^(c)^	—	—
*Mean FSMC (SD)*	43 (21.2)^(c)^	—	—
*DMD (no/first-line/second-line)*	4/6/2	—	—
**Volumetric analysis**			
*Mean WM fraction (SD) [*%*]*	41.4 (2.8)	39.0 (3.9)	0.356^(b)^
*Mean GM fraction (SD) [*%*]*	42.0 (3.2)	45.4 (3.7)	0.021^(b)^
*Mean BP fraction (SD) [*%*]*	83.3 (2.3)	84.4 (2.2)	0.166^(b)^
*Mean T2 WM lesion volume (SD) [ml]*	4.6 (5.4)	—	—

^(a)^p-value derived from Pearson’s chi-square test.

^(b)^p-values derived from Mann-Whitney test.

^(c)^no significant difference in EDSS and FSMC over time between baseline (t_1_ = 0 months) and the last follow-up (t_5_ = 12 months) using Wilcoxon signed-rank test (both p-values p > 0.05).

^(d)^first-line: glatiramer acetate or interferon-beta; second-line: natalizumab.

**SD** Standard deviation.

**EDSS** Expanded Disability Status Scale.

**FSMC** Fatigue Scale for Motor and Cognitive functions.

**WM** White matter.

**GM** Grey matter.

**BP** Brain parenchymal.

**DMD** Disease-modifying drugs.

A total of 60 fMRI data sets of MS patients and 24 fMRI data sets of the control group were analysed. There was no change of EDSS in each patient between the baseline scan and the last scan 12 months afterwards. Furthermore, we also observed no significant difference in patients’ fatigue between the baseline scan (mean FSMC 43 ± 21.2) and the final scan (mean FSMC 41 ± 22).

### No structural brain volume changes over time

To detect possible structural changes over time and to consider potential effects of these alterations on the EC measurements, we compared the MRI-derived volume measurements of the GM, WM and brain parenchyma fractions and the T2 WM lesion load for the MS patients acquired at each time point. For each of these measures, the one-way repeated-measures ANOVA showed no significant change over time (all p-values > 0.05).

### Effective connectivity changes over time

**Figure 2:**
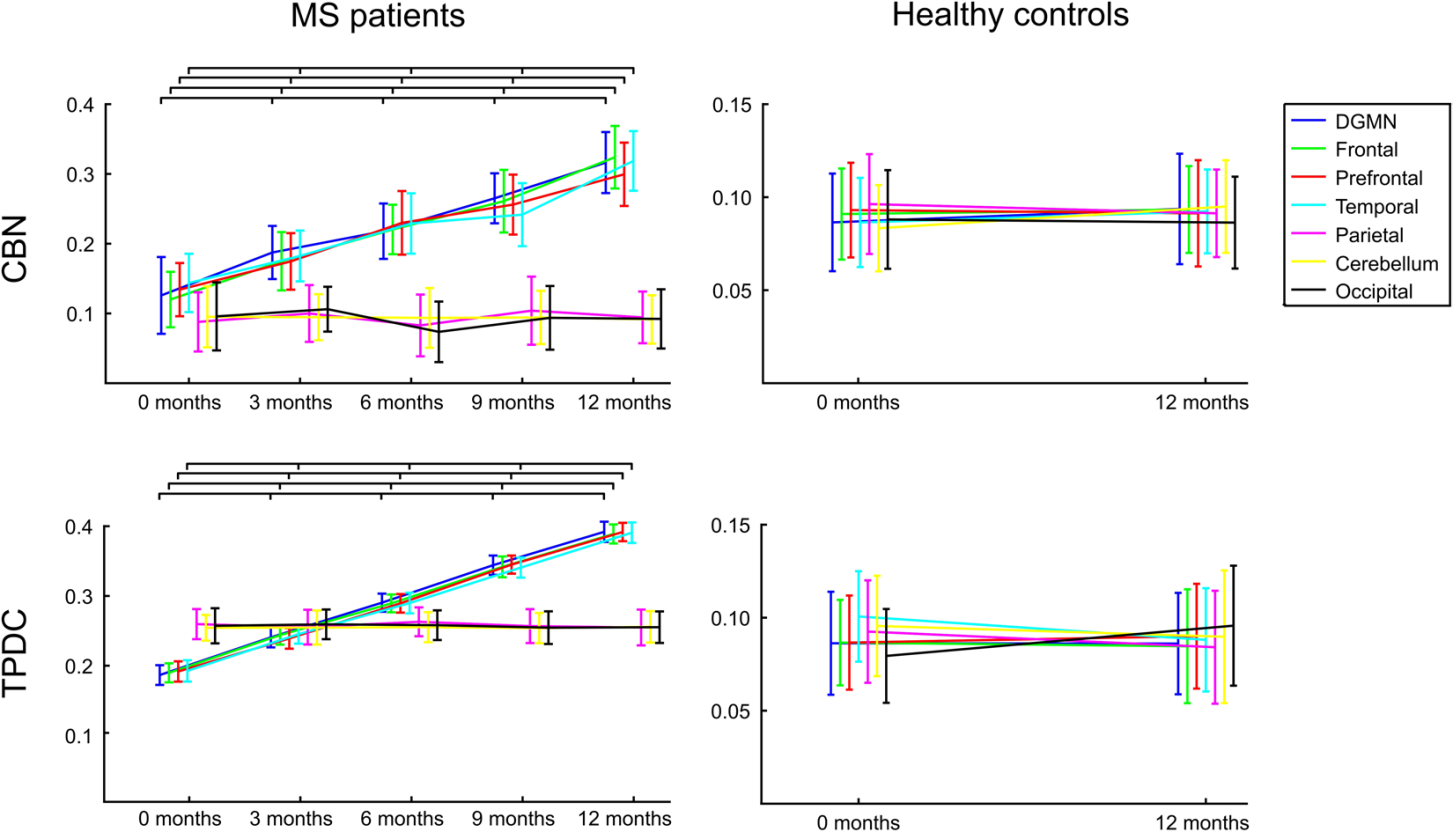
Effective connectivity analysis. Directed effective connectivity (EC) values from all MS patients at five time points showing an increase in EC in four regions: frontal, prefrontal, temporal and the deep grey matter nuclei (DGMN). EC remained unchanged in the parietal and occipital lobes and in the cerebellum over one year. EC for HC remained unchanged in both applied algorithms over the study period.

**Figure 3:**
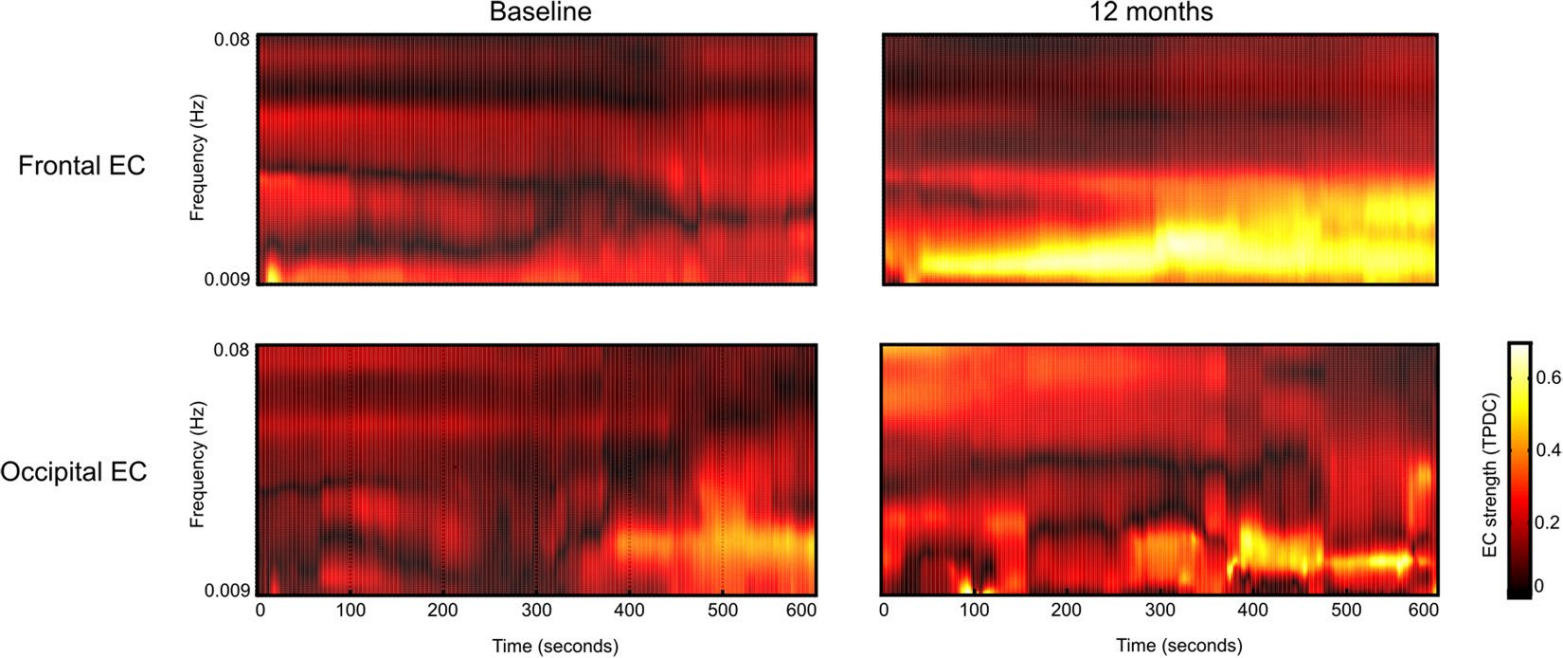
Effective connectivity analysis using Time-resolved Partial Directed Coherence (TPDC). The upper row shows one exemplary region (frontal) of increasing EC (mean of the whole patient cohort) using the TPDC analysis: The mean EC from frontal to the remaining brain regions at baseline (left) and after 12 months (right). The lower row shows an exemplary region (occipital) of unchanged EC (mean of the whole cohort) over time using the TPDC analysis: The mean EC from occipital to the remaining regions at baseline (left) and after 12 months (right).

Comparing the EC between all seven anatomical regions over time in MS patients, we observed that EC increased significantly over the 12-month study period in the frontal, prefrontal and temporal lobes as well as in the DGMN in both the CBN (Fig. 2) and the TPDC analysis (Figs. 2, 3 and S1). Conversely, the cerebellum, parietal and occipital regions showed no significant changes in EC over the five time points in either method.

For the CBN analysis, ANOVA revealed a significant effect for the factor “region” (F(6,66) = 2335, p < 0.0001). Post-hoc comparisons showed significant differences over time in the frontal, prefrontal and temporal lobes as well as in the DGMN (all post-hoc p-values < 0.001), whereas the parietal and occipital lobes and the cerebellum were not significantly different (all post-hoc p-values > 0.05). In addition, ANOVA for the factor “time” displayed a significant effect (F4,44) = 797, p < 0.0001) and post-hoc testing revealed significant differences between all comparisons (all post-hoc p-values < 0.001).

For the TPDC analysis, ANOVA showed a significant effect for the factor “region” (F(6,66) = 284, p < 0.0001). Similar to the CBN results, the post-hoc comparisons revealed significant differences over time in the frontal, prefrontal and temporal lobes as well as in the DGMN (all p-values < 0.0001), whereas the parietal and occipital lobes and the cerebellum were not significantly different (all p-values > 0.05). ANOVA for the factor “time” revealed a significant effect (F(4,44) = 3476, p < 0.0001) and post-hoc testing revealed significant differences between all comparisons (all p-values < 0.0001).

Moreover, the EC in the group of HC did not change over the complete span of 12 months in either applied method. The repeated measures ANOVA (taking into account factors “region” and “time” and “group”) revealed no significant effect for the factor “time” in the CBN analysis (F(1,22) = 2.0, p > 0.05) or in the TPDC analysis (F(1,22) = 1.0, p > 0.05), but showed a significant effect for the factor “group” in the CBN analysis (F(1,22) = 4692, p < 0.0001) as well as in the TPDC analysis (F1,22) = 2586, p < 0.0001).

Supplementary Fig. S2a highlights the dominant direction and the corresponding strength of EC at each of the five time points (originating or terminating in one of the seven brain regions) for the TPDC analysis in the MS cohort. The spatial direction pattern of the dominant EC remains almost the same over the five successive time points (slight irregularity of dominant directions only at month 6), whereas only the connections’ strength increases over time in those connections originating from or terminating in the DGMN, frontal, prefrontal or temporal regions. In contrast, HC randomly change their dominant EC direction between the two time points and do not show an increase in their EC strength over one year (Supplementary Fig. S2b). Finally, the same analysis (EC direction and strength at each of the investigated time points) using the CBN approach revealed accordant results to the TPDC analysis.

### Correlation between EC increase and EDSS, FSMC and disease duration

Based on the significant EC increase over time in four out of seven ROIs in MS patients, we calculated the difference between EC measured at baseline (t_1_ = 0 months) and the last follow up (t_5_ = 12 months), as follows: ΔEC = EC [t_5_] – EC [t_1_]. Larger values of ΔEC indicate more rapidly increasing EC. For consistency, ΔEC was only calculated for those four regions that initially showed a significant increase in EC over time.

**Figure 4:**
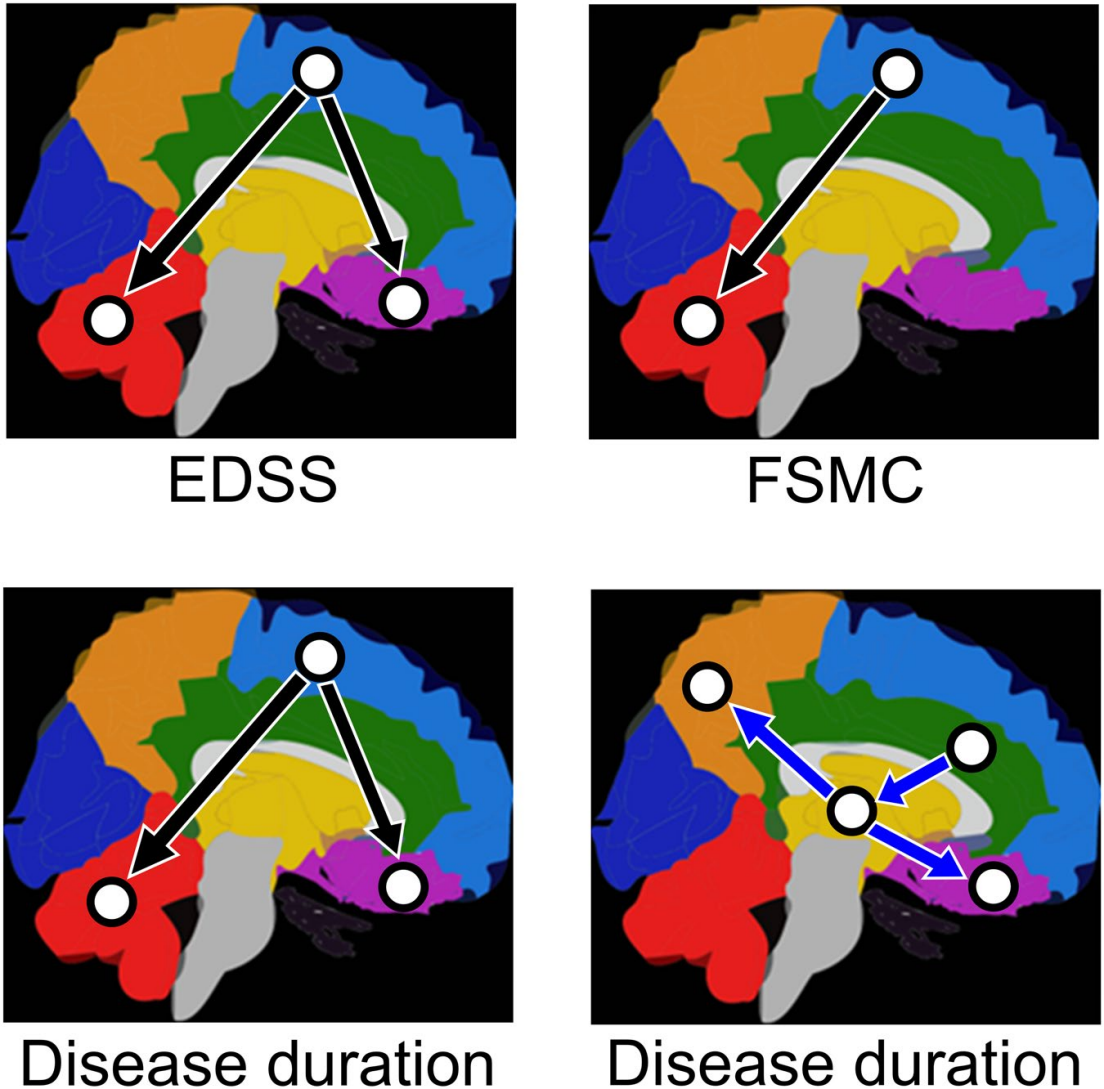
Correlation analysis. Correlation between EDSS, Fatigue Scale for Motor and Cognition (FSMC) or disease duration and effective connectivity (EC) values from the MS patients between baseline (t_1_ = 0 months) and the last follow-up (t_5_ = 12 months) calculated as ΔEC = EC [t_5_] − EC [t_1_]. Only significant correlations between ΔEC and EDSS, FSMC or disease duration are highlighted. Black arrows indicate inverse (negative) correlations and blue arrows indicate direct (positive) correlations. Purple prefrontal. Light blue frontal. Orange parietal. Dark blue occipital. Green temporal. Red cerebellum. Yellow DGMN.

The correlation analyses of ΔEC with EDSS and FSMC values at baseline both showed similar correlations (Table 2 and Figs. 4 and S3). Precisely, the ΔEC from frontal to prefrontal was inversely correlated with EDSS (for CBN: r = −0.58, p = 0.0003 and for TPDC: r = −0.71, p = 0.0004) as was the ΔEC from frontal to the cerebellum (for CBN: r = −0.52, p = 0.0007 and for TPDC: r = −0.65, p = 0.0002). The ΔEC from the frontal region to the cerebellum (for CBN: r = −0.54, p = 0.0005 and for TPDC: r = −0.62, p = 0.008) showed an inverse correlation with the FSMC. These inverse correlations with both EDSS and FSMC imply that less physical disability and less fatigue was observed in MS patients with higher ΔEC values.

**Table 2:** Correlation between (a) Expanded Disability Status Scale (EDSS), Fatigue Scale for Motor and Cognitive functions (FSMC) or disease duration and effective connectivity (EC) values between baseline (t_1_ = 0 months) and the last follow-up (t_5_ = 12 months) calculated as ΔEC = EC [t_5_] − EC [t_1_]. Higher ΔEC values correspond to more rapidly increasing EC. Only significant directed connections from the deep grey matter nuclei (DGMN), frontal, prefrontal and temporal regions were considered in the correlation analyses between ΔEC and the three clinical parameters.

Correlation with …	ΔEC from …	To …	CBN analysis		TPDC analysis	
			Pearson’s *r*	p-value^(a)^	Pearson’s r	p-value^(a)^
EDSS	frontal	prefrontal	−0.58	*0.0003*	−0.71	*0.0004*
	frontal	cerebellum	−0.52	*0.0007*	−0.65	*0.0002*
FSMC	frontal	cerebellum	−0.54	*0.0005*	−0.62	*0.0080*
Disease duration	frontal	prefrontal	−0.50	*0.0008*	−0.58	*0.0003*
	frontal	cerebellum	−0.69	*0.0006*	−0.77	*0.0008*
	DGMN	prefrontal	0.62	*0.0005*	0.54	*0.0005*
	DGMN	parietal	0.67	*0.0004*	0.59	*0.0007*
	temporal	DGMN	0.66	*0.0028*	0.58	*0.0064*

**EDSS** Expanded Disability Status Scale.

**FSMC** Fatigue Scale for Motor and Cognitive functions.

**EC** Effective connectivity.

**CBN** Causal Bayesian Networks.

TPDC Time-resolved Partial Directed Coherence.

Bonferroni’s corrected p-values.

The correlation analysis with patients’ disease duration revealed both positive and negative correlations. ΔEC between the frontal and prefrontal regions (for CBN: r = −0.50, p = 0.0008 and for TPDC: r = −0.58, p = 0.0003) as well as between the frontal region and cerebellum (for CBN: r = −0.69, p = 0.0006 and for TPDC: r = −0.77, p = 0.0008) revealed an inverse (negative) correlation with disease duration. This suggests that ΔEC is higher with shorter disease duration (time since the first clinical event). However, positive correlations with disease duration were found in other regions: temporal to the DGMN (for CBN: r = 0.66, p = 0.0028 and for TPDC: r = 0.58, p = 0.0064), DGMN to prefrontal (for CBN: r = 0.62, p = 0.0005 and for TPDC: r = 0.54, p = 0.0005) and DGMN to parietal (for CBN: r = 0.67, p = 0.0004 and for TPDC: r = 0.59, p = 0.0007). For an overview of the mathematical results of both methods please see Table 2 and Supplementary Fig. S4, and by way of illustration refer to simplified Fig. 4.

## Discussion

Our findings provide evidence that EC in patients with clinically stable MS changes markedly over a short period of time with a distinct topographical specificity. Whereas frontal, prefrontal and temporal lobes as well as the deep grey matter exhibited an increase in EC to all other regions over time, EC values for the parietal and occipital lobes and the cerebellum remained unchanged. Moreover, for all investigated regions, EC did not change in HC over the same period of time. Furthermore, clinical disability at study entrance (described through the EDSS) inversely correlated with an increase in EC over time from the frontal and prefrontal cortex to the cerebellum. In addition, the increase in EC from the frontal lobe to the cerebellum was inversely correlated to patients’ fatigue (described through the FSMC).

These identified longitudinal EC data provide novel insights into the recruitment of directed connections in MS patients over time, by showing for the first time where and when functional reorganization processes occur in the absence of relapses, disease progression and MRI activity. Our observed inverse correlation between EC and clinical markers for disability (EDSS and FSMC) could advocate for EC being a surrogate marker of patients’ functional reserve capacity and adaptability despite chronic neuroinflammation.

So far, there have been only few studies analysing EC in patients with MS. A large cohort with a wide range of disabilities and disease durations was investigated in a multi-center fMRI study by Rocca *et al*.^10^. The authors found increased EC in regions of the sensorimotor network in MS patients compared to HC. They postulated that the enhancement of directed information flow may provide an important compensatory mechanism in MS. Increased functional connectivity at rest (mainly temporal interactions between interconnected regions) in the motor network was also shown in patients with clinically isolated syndrome and early MS in comparison to HC^37,38^.

In the present study, we address EC changes longitudinally. We suggest that cortical reorganization occurs continuously in remission phases and in the absence of disease progression as an important adaptive mechanism to maintain sensorimotor and cognitive function. However, the EC increase may be an early phenomenon of cortical adaptation, which potentially abates as the disease progresses, as indicated by diffusely altered connectivity and abnormal network function in studies examining later stages of the disease^39,40^ as well as the loss and reversal of connections in progressive MS^14^.

Although we examined EC at rest, our findings are consistent with the so-called neuronal efficiency hypothesis, which implies that greater involvement, particularly of prefrontal areas, is required to maintain network performance^41^. We show that the prefrontal and frontal lobe connections continuously strengthen over a short time in patients with RRMS. This might be a consequence of functional compensatory mechanisms preserving good clinical performance despite diffuse inflammatory activity in the normal-appearing tissue substantiated by the lack of clinical progression and relapses in our patient cohort. It was previously reported that additional recruitment of (medial) prefrontal regions is essential for the maintenance of cognitive function in MS patients, as shown in a functional connectivity analysis of a Go/No-go task with increasing complexity^42^.

We note that although our patient population was longitudinally investigated over several time points, the small sample size may have affected our ability to reveal more subtle effects due to the limited statistical power. However, cross-sectional studies cannot differentiate between reorganization processes driven by either an acute inflammatory attack or chronic tissue damage. We have recently shown that functional network changes in MS patients with chronic lesions occur as a result of reorganization processes following the initial appearance of an acute lesion^43^. Here, we highlight that such continuous reorganization processes proceed in the absence of acute inflammatory attacks (clinical and subclinical) by providing information about directed interactions between brain regions.

Furthermore, an abnormal activation pattern and EC alterations between the right cerebellum and fronto-parietal areas were previously observed in MS patients and were associated with a better performance during an interference task^44^. Our longitudinal findings emphasize that an increase in EC at rest between the frontal lobe and the cerebellum is already detectable over a relatively short period of time. The inverse correlation between the connectivity strength of these directed connections and EDSS/fatigue suggests that the frontal lobe causally influences the cerebellum by increasing connections to the cerebellum. Hence, the fronto-cerebellar EC increase seems to be involved in the maintenance of clinical performance. The cerebellum is part of several large-scale networks involving extended parts of the neocortex including motor and association areas in the frontal lobe that are known for their contributions to motor and higher cognitive functions in MS patients^45,46^.

The observed inverse correlations with clinical disability, assessed by EDSS and FSMC, suggest that the reorganization detected at rest is important in the maintenance of adequate functioning, as mentioned above. Another interpretative approach of this phenomenon is that the least impaired patients have the largest capacity for increasing EC, suggesting that patients with low EDSS and low FSMC provide better adaptive compensatory mechanisms. A strengthening of information flows originating in the frontal lobe and reaching the cerebellum is associated with lower EDSS and FSMC values. This is supported by early fMRI studies which suggested that cerebellar–cortical functional connectivity mediates adaptive transformations in brain motor control circuits in patients with MS^47^.

Significant alterations of the fronto-cerebellar connections were replicated in all three correlation analyses, namely with EDSS, FSMC and disease duration, as well as through both applied methodological approaches. The predominance of the fronto-cerebellar connectivity might indicate that, even in the absence of task performances, the recruitment of connections within this pathway is relevant for sustaining functional integrity or even preventing functional decline. The cerebellum is critically involved in the temporo-spatial integration of neural inputs from descending cortico-cerebellar projections^45^ and serves as a control center for motor function as well as higher order cognitive functions^48^. The observed inverse correlation of increasing fronto-cerebellar EC with clinical disability points towards an improvement in top–down control processes as an adaptive role in limiting clinical consequences in early RRMS patients.

In comparison to the EDSS and FSMC correlation analysis, we could see a similar pattern in the correlation analysis of EC with disease duration. More precisely, higher values of EC from frontal to prefrontal lobe and from the frontal lobe to the cerebellum were associated with shorter disease durations. In turn, this illustrates that patients with longer disease duration exhibited only a minor increase of EC in these regions. Despite this observation, it should be considered that there were also positive correlations between disease duration and EC (DGMN to prefrontal, DGMN to parietal and temporal to the DGMN). This demonstrates that compared to EDSS and FSMC the association with disease duration is more heterogeneous and reveals similar but also distinct patterns of cortical activation. It indicates that in the early stages of the disease the fronto-prefrontal as well as the fronto-cerebellar connections underlie a dynamic reorganization, whereas the recruitment of connections originating in the DGMN and the temporal lobe presumably dominate in the later stages of the disease. This heterogeneity of directed connections in association with the disease duration of the patients is in line with a recent study observing distinct EC patterns among various subtypes of MS patients^13^.

The modality fMRI has always been used for assessing connectivity over extended periods of time, however, nowadays it is becoming clear that it can be used to resolve temporal dynamics of functional or effective connectivity^49,50^. Functional MRI data are poorly sampled in time, but are still capable of capturing dynamical events as in the electrophysiological domain^51^. In most of the connectivity analyses using autoregressive processes as the basis, the systems are analysed with static MVAR coefficients as it is done in CBN analyses, i.e., the fitted model and MVAR coefficients remain the same for the complete length of the data^52,53^. However, the fitted model should be time-varying for non-linear systems like functional data^54^ from the brain as in our study. For such adaptive models, the corresponding coefficients need to be estimated regularly over the course of time. These adaptive models are particularly useful for modelling non-linear processes, where autoregressive coefficients and the variance of the process are allowed to change over the course of time^55^. Despite these advantages of using TPDC over other (more conventional) causality methods like CBN, one drawback is that TPDC is time-consuming, owing to the estimation of MVAR coefficients at each time point, which limits its usage depending on the available computational resources^21^. However, despite this, our findings lend support to the increased application of this analysis where possible, especially over longer time periods.

Finally, one further methodological consideration relates to the fact that the time series were extracted from different regions of the same lobe (selection of large-scale ROIs) and thus can potentially show different shapes and behaviours over the course of the acquisition. However, here our ultimate focus was not on a high spatial specificity, but rather on a conceptual overview of longitudinal EC dynamics in MS patients achieving maximal population-level reproducibility (i.e. as extracted from anatomical atlases and usable for the clinical practice) and biological validity while also accommodating individual anatomic variability.

In conclusion, our data show longitudinal region-specific increases in EC in resting-state fMRI in patients with clinically stable MS. Fronto-cerebellar connections might play an important role in counteracting continuous damage resulting from demyelination and neuronal injury, thereby maintaining brain function in MS. We believe that these findings are encouraging and should be further investigated in larger patient cohorts as well as over longer observation periods.

## Supplementary information

Supplementary information.

## Data Availability

The datasets analysed during this study are available from the corresponding author on request.
