# Supplementary material for: Continuous reorganization of cortical information flow in multiple sclerosis: A longitudinal fMRI effective connectivity study
Source: Sci Rep. 2020 Jan 21;10:806. doi: 10.1038/s41598-020-57895-x (PMC6972853; doi:10.1038/s41598-020-57895-x)
Supplement: Supplementary file 1 — Supplementary information. [file 41598_2020_57895_MOESM1_ESM.pdf]

Continuous reorganization of cortical information flow in multiple sclerosis: A longitudinal fMRI effective connectivity study

Vinzenz Fleischer, MD[1#], Muthuraman Muthuraman, PhD[1#], Abdul Rauf Anwar, PhD[2], Gabriel Gonzalez-Escamilla, PhD[1], Angela Radetz, MSc[1], René-Maxime Gracien, MD[3], Stefan Bittner, MD[1], Felix Lüssi, MD[1], Sven G. Meuth, PhD, MD[4], Frauke Zipp, MD[1§] and Sergiu Groppa, MD[1§*]

Supplement

Supplementary Table S1. Regions of interest included in the AAL-atlas and their affiliation to the seven investigated superordinate brain areas (merged regions) in the current study.

Superordinate brain region	AAL-based brain regions	Abbreviation
**DGMN (8)**		
	Caudate nucleus (left)	Caudate_L
	Caudate nucleus (right)	Caudate_R
	Lenticular nucleus, putamen (left)	Putamen_L
	Lenticular nucleus, putamen (right)	Putamen_R
	Lenticular nucleus, putamen (left)	Pallidum_L
	Lenticular nucleus, putamen (right)	Pallidum_R
	Thalamus (left)	Thalamus_L
	Thalamus (right)	Thalamus_R
**Frontal (22)**		
	Precentral gyrus (left)	Precentral_L
	Precentral gyrus (right)	Precentral_R
	Superior frontal gyrus, dorsolateral (left)	Frontal_Sup_L
	Superior frontal gyrus, dorsolateral (right)	Frontal_Sup_R
	Middle frontal gyrus (left)	Frontal_Mid_L
	Middle frontal gyrus (right)	Frontal_Mid_R
	Middle frontal gyrus, orbital part (left)	Frontal_Mid_Orb_L
	Middle frontal gyrus, orbital part (right)	Frontal_Mid_Orb_R
	Inferior frontal gyrus, opercular part (left)	Frontal_Inf_Oper_L
	Inferior frontal gyrus, opercular part (right)	Frontal_Inf_Oper_R
	Inferior frontal gyrus, triangular part (left)	Frontal_Inf_Tri_L
	Inferior frontal gyrus, triangular part (right)	Frontal_Inf_Tri_R

	Inferior frontal gyrus, orbital part (left)	Frontal_Inf_Orb_L
	Inferior frontal gyrus, orbital part (right)	Frontal_Inf_Orb_R
	Gyrus rectus (left)	Rectus_L
	Gyrus rectus (right)	Rectus_R
	Rolandic operculum (left)	Rolandic_Oper_L
	Rolandic operculum (left)	Rolandic_Oper_R
	Supplementary motor area (left)	Supp_Motor_Area_L
	Supplementary motor area (right)	Supp_Motor_Area_R
	Superior frontal gyrus, medial orbital (left)	Frontal_Med_Orb_L
	Superior frontal gyrus, medial orbital (right)	Frontal_Med_Orb_R
**Prefrontal (4)**		
	Superior frontal gyrus, orbital part (left)	Frontal_Sup_Orb_L
	Superior frontal gyrus, orbital part (right)	Frontal_Sup_Orb_R
	Superior frontal gyrus, medial (left)	Frontal_Sup_Medial_L
	Superior frontal gyrus, medial (right)	Frontal_Sup_Medial_R
**Temporal (22)**		
	Heschl gyrus (left)	Heschl_L
	Heschl gyrus (right)	Heschl_R
	Superior temporal gyrus (left)	Temporal_Sup_L
	Superior temporal gyrus (right)	Temporal_Sup_R
	Temporal pole: superior temporal gyrus (left)	Temporal_Pole_Sup_L
	Temporal pole: superior temporal gyrus (right)	Temporal_Pole_Sup_R
	Middle temporal gyrus (left)	Temporal_Mid_L
	Middle temporal gyrus (right)	Temporal_Mid_R
	Temporal pole: middle temporal gyrus (left)	Temporal_Pole_Mid_L
	Temporal pole: middle temporal gyrus (right)	Temporal_Pole_Mid_R
	Inferior temporal gyrus (left)	Temporal_Inf_L
	Inferior temporal gyrus (right)	Temporal_Inf_R
	Hippocampus (left)	Hippocampus_L
	Hippocampus (right)	Hippocampus_R

	Parahippocampal gyrus (left)	ParaHippocampal_L
	Parahippocampal gyrus (right)	ParaHippocampal_R
	Amygdala (left)	Amygdala_L
	Amygdala (right)	Amygdala_R
	Insula (left)	Insula_L
	Insula (right)	Insula_R
	Olfactory cortex (left)	Olfactory_L
	Olfactory cortex (right)	Olfactory_R
**Parietal (20)**		
	Fusiform gyrus (left)	Fusiform_L
	Fusiform gyrus (right)	Fusiform_R
	Postcentral gyrus (left)	Postcentral_L
	Postcentral gyrus (right)	Postcentral_R
	Superior parietal gyrus (left)	Parietal_Sup_L
	Superior parietal gyrus (right)	Parietal_Sup_R
	Inferior parietal, but supramarginal and angular gyri (left)	Parietal_Inf_L
	Inferior parietal, but supramarginal and angular gyri (right)	Parietal_Inf_R
	Supramarginal gyrus (left)	SupraMarginal_L
	Supramarginal gyrus (right)	SupraMarginal_R
	Angular gyrus (left)	Angular_L
	Angular gyrus (right)	Angular_R
	Precuneus (left)	Precuneus_L
	Precuneus (right)	Precuneus_R
	Paracentral lobule (left)	Paracentral_Lobule_L
	Paracentral lobule (right)	Paracentral_Lobule_R
	Cuneus (left)	Cuneus_L
	Cuneus (right)	Cuneus_R
	Lingual gyrus (left)	Lingual_L
	Lingual gyrus (right)	Lingual_R

**Occipital (14)**		
	Anterior cingulate and paracingulate gyri (left)	Cingulum_Ant_L
	Anterior cingulate and paracingulate gyri (right)	Cingulum_Ant_R
	Median cingulate and paracingulate gyri (left)	Cingulum_Mid_L
	Median cingulate and paracingulate gyri (right)	Cingulum_Mid_R
	Posterior cingulate gyrus (left)	Cingulum_Post_L
	Posterior cingulate gyrus (right)	Cingulum_Post_R
	Calcarine fissure and surrounding cortex (left)	Calcarine_L
	Calcarine fissure and surrounding cortex (right)	Calcarine_R
	Superior occipital gyrus (left)	Occipital_Sup_L
	Superior occipital gyrus (right)	Occipital_Sup_R
	Middle occipital gyrus (left)	Occipital_Mid_L
	Middle occipital gyrus (right)	Occipital_Mid_R
	Inferior occipital gyrus (left)	Occipital_Inf_L
	Inferior occipital gyrus (right)	Occipital_Inf_R
**Cerebellum (26)**		
	Crus I of cerebellar hemisphere (left)	Cerebelum_Crus1_L
	Crus I of cerebellar hemisphere (right)	Cerebelum_Crus1_R
	Crus II of cerebellar hemisphere (left)	Cerebelum_Crus2_L
	Crus II of cerebellar hemisphere (right)	Cerebelum_Crus2_R
	Lobule III of cerebellar hemisphere (left)	Cerebelum_3_L
	Lobule III of cerebellar hemisphere (right)	Cerebelum_3_R
	Lobule IV/V of cerebellar hemisphere (left)	Cerebelum_4_5_L
	Lobule IV/V of cerebellar hemisphere (right)	Cerebelum_4_5_R
	Lobule VI of cerebellar hemisphere (left)	Cerebelum_6_L
	Lobule VI of cerebellar hemisphere (right)	Cerebelum_6_R
	Lobule VIIb of cerebellar hemisphere (left)	Cerebelum_7b_L
	Lobule VIIb of cerebellar hemisphere (right)	Cerebelum_7b_R
	Lobule VIII of cerebellar hemisphere (left)	Cerebelum_8_L
	Lobule VIII of cerebellar hemisphere (right)	Cerebelum_8_R

	Lobule IX of cerebellar hemisphere (left)	Cerebelum_9_L
	Lobule IX of cerebellar hemisphere (right)	Cerebelum_9_R
	Lobule X of cerebellar hemisphere (left)	Cerebelum_10_L
	Lobule X of cerebellar hemisphere (right)	Cerebelum_10_R
	Lobule I/II of vermis	Vermis_1_2
	Lobule III of vermis	Vermis_3
	Lobule IV/V of vermis	Vermis_4_5
	Lobule VI of vermis	Vermis_6
	Lobule VII of vermis	Vermis_7
	Lobule VIII of vermis	Vermis_8
	Lobule IX of vermis	Vermis_9
	Lobule X of vermis	Vermis_10

Supplementary Figure legends

Supplementary Figure S1. Illustration of the EC dynamics using the TPDC approach in (A) MS patients and (B) healthy controls.

The EC strength dynamic over the acquired resting-state time (600ms) is shown for each of the investigated regions for MS patients and healthy controls, separately.

In MS patients (A) the upper row shows the EC strength dynamic originating from the DGMN, frontal, prefrontal, temporal, parietal and occipital region as well as the cerebellum (arrow on the right side), whereas the lower row shows the EC strength dynamic terminating in the above-mentioned regions (arrow on the left side).

In healthy controls (B), the upper row shows accordingly the EC strength dynamic originating from the seven investigated regions (arrow to the right side), whereas the lower row shows the EC strength dynamic terminating in the seven investigated regions (arrow on the left side).

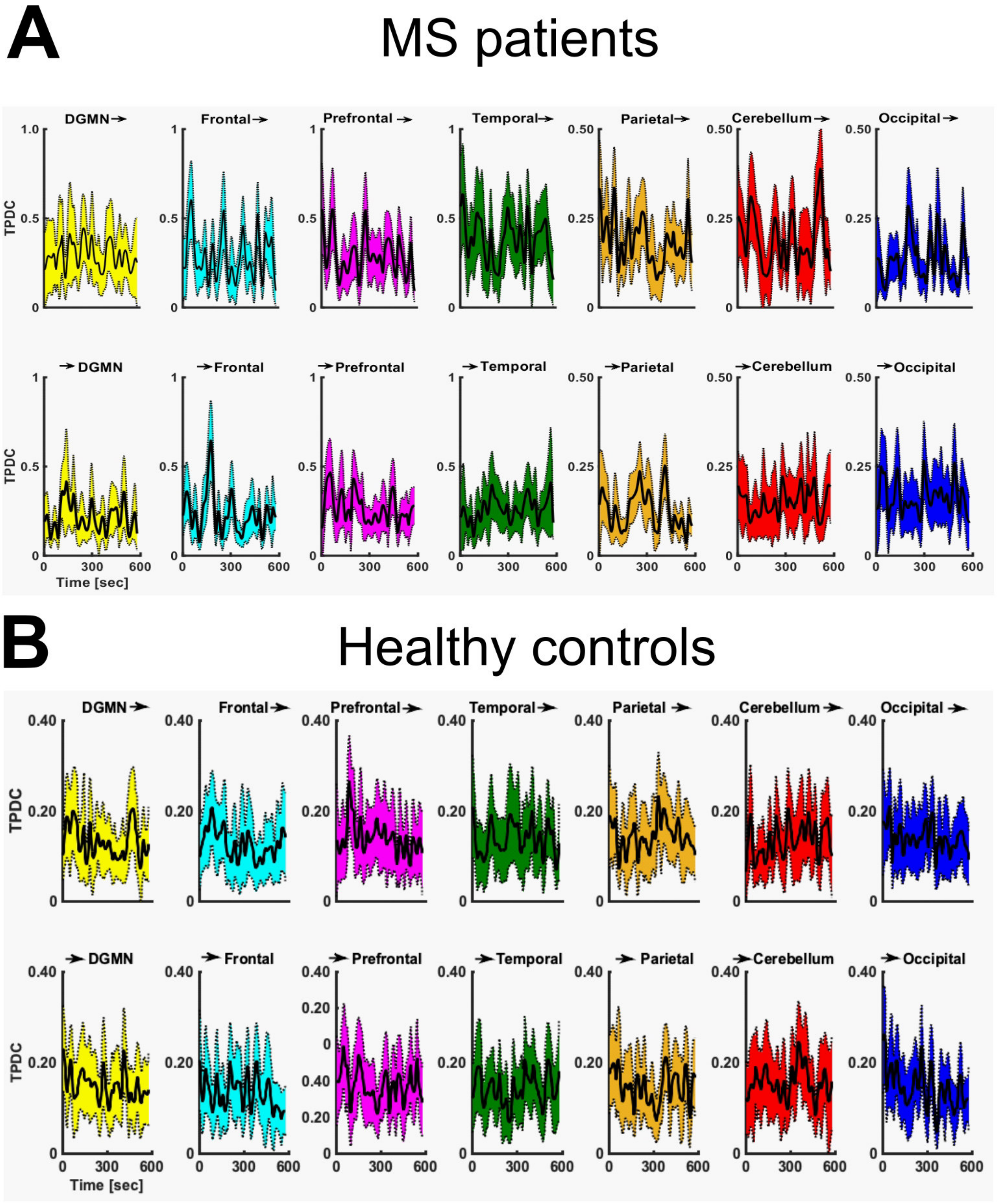
A
MS patients
DGMN→
Frontal→
Prefrontal→
Temporal→
Parietal→
Cerebellum→
Occipital→
→DGMN
→Frontal
→Prefrontal
→Temporal
→Parietal
→Cerebellum
→Occipital
TPDC
Time [sec]
B
Healthy controls
DGMN→
Frontal→
Prefrontal→
Temporal→
Parietal→
Cerebellum→
Occipital→
→DGMN
→Frontal
→Prefrontal
→Temporal
→Parietal
→Cerebellum
→Occipital
TPDC
Time [sec]

Supplementary Figure S2a and S2b. The dominant direction and its strength of EC at each time point for MS patients (A) and healthy controls (B).

The dominant direction (indicated by arrows) and its strength (indicated by different colors) for each time point is shown for each of the seven investigated regions – illustrated separately for MS patients (A) and healthy controls(B).

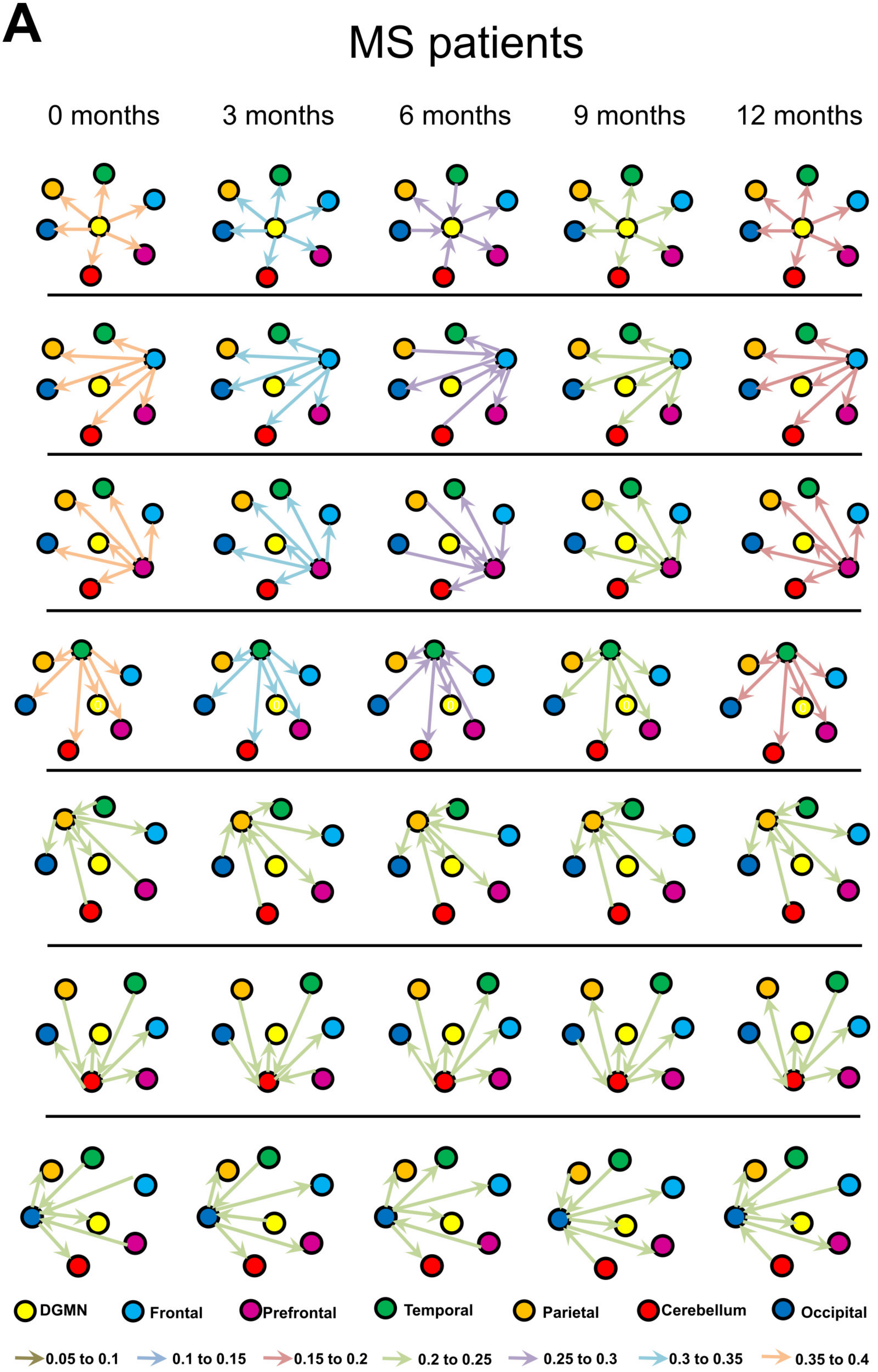

A
MS patients
0 months
3 months
6 months
9 months
12 months
DGMN
Frontal
Prefrontal
Temporal
Parietal
Cerebellum
Occipital
0.05 to 0.1
0.1 to 0.15
0.15 to 0.2
0.2 to 0.25
0.25 to 0.3
0.3 to 0.35
0.35 to 0.4

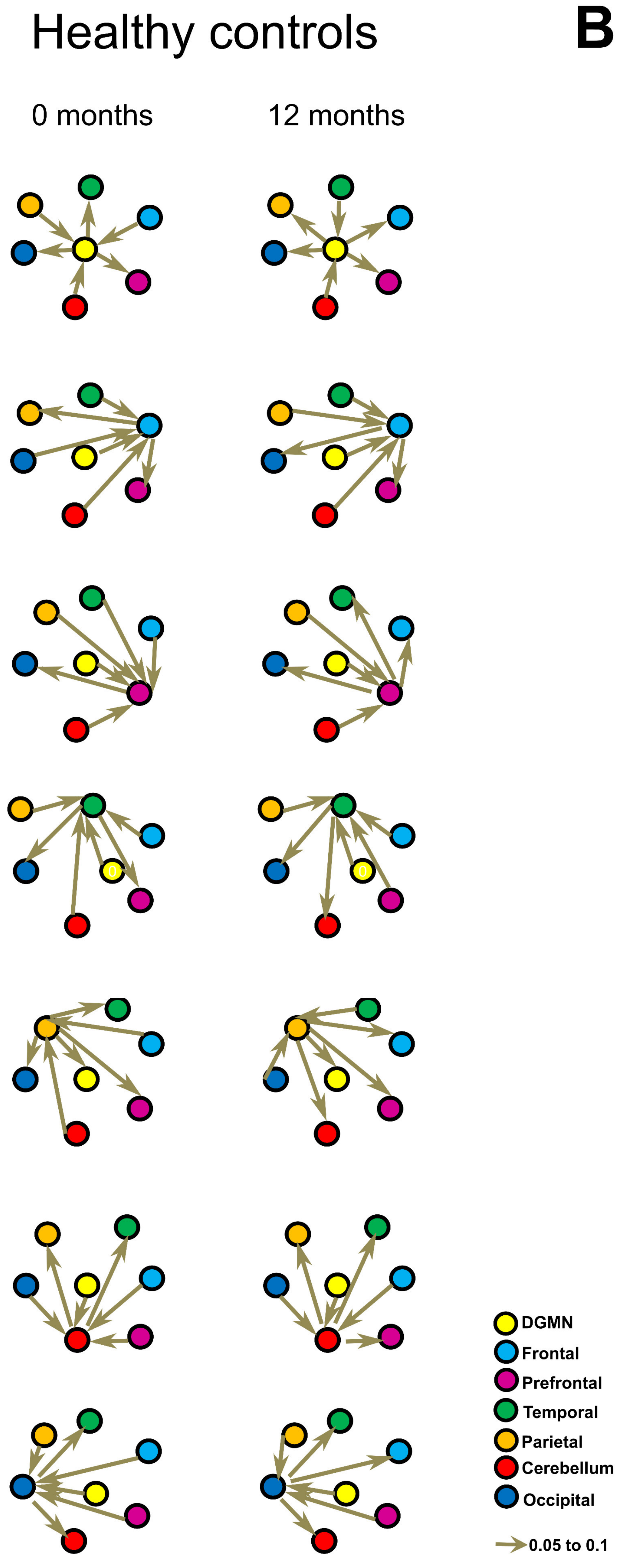

Healthy controls
B
0 months
12 months
0
0
DGMN
Frontal
Prefrontal
Temporal
Parietal
Cerebellum
Occipital
0.05 to 0.1

Supplementary Figure S3. The correlation analysis with clinical parameters performed with (A) the difference between the baseline and last follow-up and with (B) the standard deviation of EC over time.

The correlation analysis on the left side (A) corresponds to Figure 4 of the main text and shows the correlation between EDSS, FSMC or disease duration and EC values from MS patients between baseline (t_1 = 0 months) and the last follow-up (t_5 = 12 months) calculated as $\Delta EC = EC [t_5] - EC [t_1]$.

The illustration on the right side (B) shows the correlation analysis using the standard deviation of EC over time instead of the above-mentioned approach. Only the positive correlation with disease duration from the temporal region to the DGMN did not reach significance level compared to the approach using the difference between the baseline and last follow-up. The remaining correlation pattern appeared unchanged. This was shown for the EC values derived from both the CBN as well as TPDC analysis.

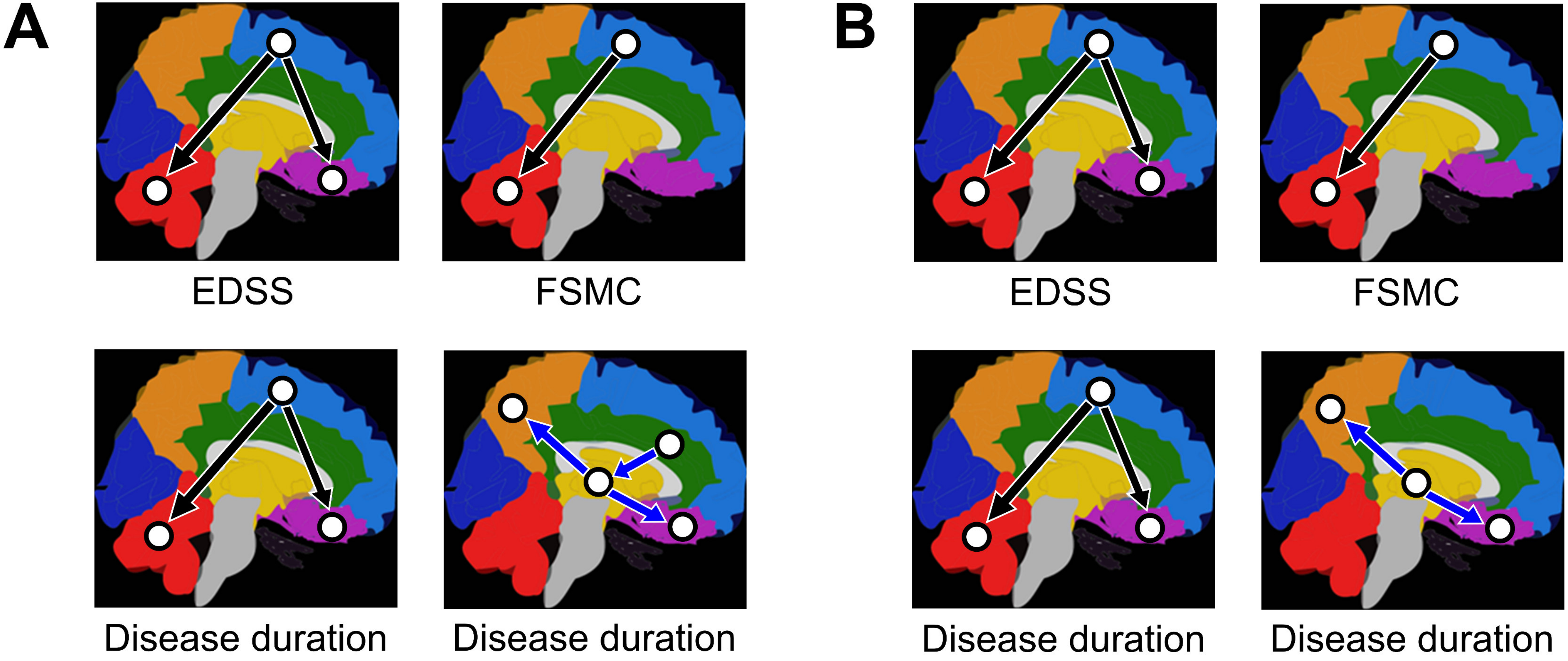
A
EDSS
FSMC
Disease duration
Disease duration
B
EDSS
FSMC
Disease duration
Disease duration

Supplementary Figure 4. The scatterplots of the correlation analysis between the clinical parameters and the individual effective connectivity (EC) changes illustrated separately for the CBN (A) and TPDC analysis (B).

Correlation between the Expanded Disability Status Scale (EDSS), Fatigue Scale for Motor and Cognitive functions (FSMC) or disease duration and effective connectivity (EC) values between baseline (t_1 = 0 months) and the last follow-up (t_5 = 12 months) calculated as ΔEC = EC [t_5] – EC [t_1]. The corresponding values (correlation coefficients and p-values) are shown in Table 2.

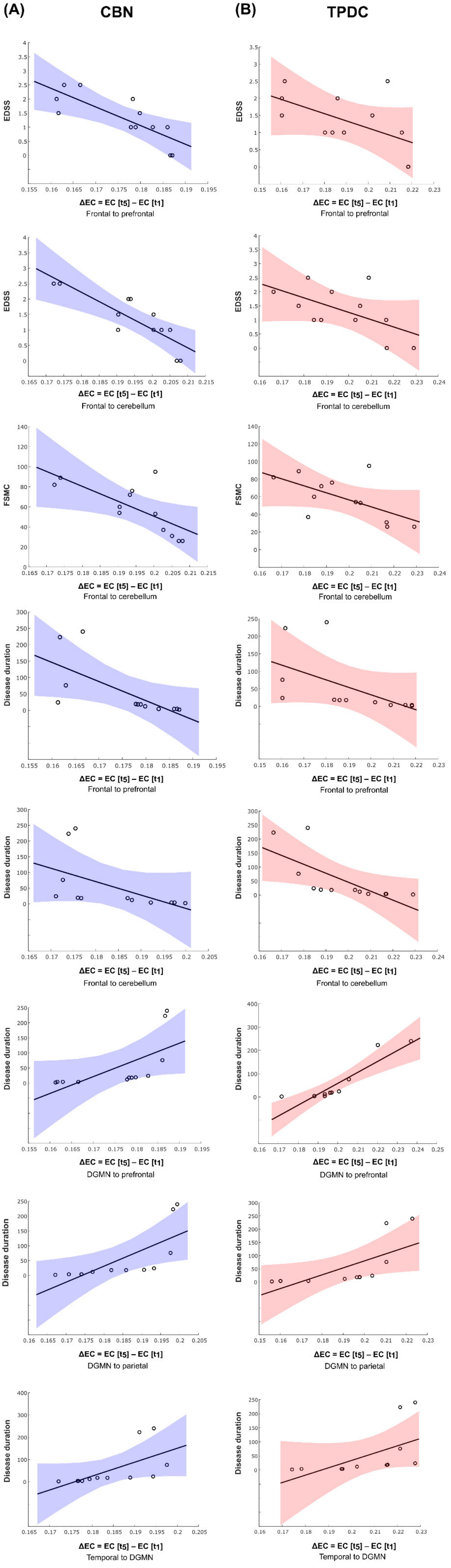

(A)
CBN
(B)
TPDC
EDSS
ΔEC = EC [t5] – EC [t1]
Frontal to prefrontal
EDSS
ΔEC = EC [t5] – EC [t1]
Frontal to prefrontal
EDSS
ΔEC = EC [t5] – EC [t1]
Frontal to cerebellum
EDSS
ΔEC = EC [t5] – EC [t1]
Frontal to cerebellum
FSMC
ΔEC = EC [t5] – EC [t1]
Frontal to cerebellum
FSMC
ΔEC = EC [t5] – EC [t1]
Frontal to cerebellum
Disease duration
ΔEC = EC [t5] – EC [t1]
Frontal to prefrontal
Disease duration
ΔEC = EC [t5] – EC [t1]
Frontal to prefrontal
Disease duration
ΔEC = EC [t5] – EC [t1]
Frontal to cerebellum
Disease duration
ΔEC = EC [t5] – EC [t1]
Frontal to cerebellum
Disease duration
ΔEC = EC [t5] – EC [t1]
DGMN to prefrontal
Disease duration
ΔEC = EC [t5] – EC [t1]
DGMN to prefrontal
Disease duration
ΔEC = EC [t5] – EC [t1]
DGMN to parietal
Disease duration
ΔEC = EC [t5] – EC [t1]
DGMN to parietal
Disease duration
ΔEC = EC [t5] – EC [t1]
Temporal to DGMN
Disease duration
ΔEC = EC [t5] – EC [t1]
Temporal to DGMN
